# Distinct Effects of Biological Treatments on Eosinophils and Neutrophils in Chronic Rhinosinusitis With Nasal Polyp Patients

**DOI:** 10.1002/clt2.70117

**Published:** 2025-11-27

**Authors:** Sharon Van Nevel, Nan Zhang, Zhaofeng Xu, Manon Blauwblomme, Elke Vandewalle, Gabriele Holtappels, Natalie De Ruyck, Filip Van Nieuwerburgh, Stijn Vanhee, Philippe Gevaert, Claus Bachert

**Affiliations:** ^1^ Upper Airways Research Laboratory Department of Head and Skin Faculty of Medicine Ghent University Ghent Belgium; ^2^ Department of Otorhinolaryngology The First Affiliated Hospital of Sun Yat‐sen University International Airways Research Center Sun Yat‐sen University Guangzhou China; ^3^ NXTGNT Laboratory, Department of Pharmaceutics Ghent University Ghent Belgium; ^4^ Department of Otorhinolaryngology, Head and Neck Surgery University of Münster Münster Germany

**Keywords:** biological treatments, chronic rhinosinusitis with nasal polyps, eosinophilic inflammation, neutrophilic inflammation

## Abstract

**Background:**

Chronic rhinosinusitis with nasal polyps (CRSwNP) is generally characterized by tissue‐infiltrating eosinophils. Various biologic treatments, targeting the inflammation, have demonstrated efficacy in reducing nasal polyp size and symptoms. However, their specific impact on granulocyte populations within polyps remains largely unclear. This study explores how different biological treatments modulate local nasal polyp inflammation by assessing changes in granulocyte presence and recruitment before and after treatment.

**Methods:**

Type 2‐high CRSwNP patients received treatment with mepolizumab, benralizumab, omalizumab, or dupilumab. Immunohistochemistry and protein measurements were performed on their nasal polyp tissue. Bulk RNA‐sequencing was conducted on pre‐ and post‐treatment nasal samples, identifying differentially expressed genes. These results were integrated with single‐cell data from CRSwNP patients.

**Results:**

Nasal polyp tissue from type 2‐high patients exhibited substantial eosinophil infiltration and limited neutrophils present. All tested biologics reduced eosinophil‐related proteins and genes in nasal tissue. However, our data suggest that benralizumab, mepolizumab and omalizumab could induce a concurrent upregulation of neutrophilic markers. In these patients, chemoattractant genes for neutrophils primarily originated from the epithelial cell cluster, whereas receptors for these biologics were expressed by plasma cells, dendritic cells, and mast cells.

**Conclusion:**

Biological treatments effectively reduced eosinophilic inflammation in nasal polyps. However, most biologics could induce an eosinophil‐to‐neutrophil shift, indicating that solely targeting eosinophils may be insufficient as a treatment approach. Understanding these secondary effects on local immune pathways is critical for optimizing CRSwNP treatment strategies.

## Graphical abstract

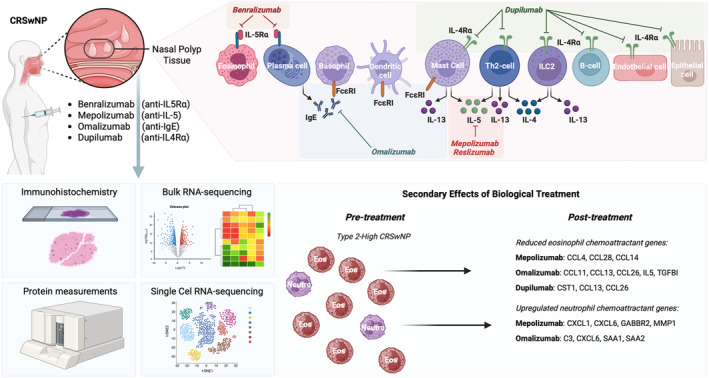

## 1 Introduction

CRSwNP is a prevalent condition in Europe, affecting 2.1%–4.3% of the population [1, 2, 3], with patients suffering from a persistent inflammation within the nasal and sinus mucosa, in combination with polyp formation [4]. Approximately 85% of these patients exhibit a type 2‐high inflammation, with elevated levels of IL‐5, IgE, and eosinophil cationic protein (ECP) within the nasal polyp tissue, coupled with eosinophil infiltration [5, 6]. These eosinophils are associated with disease severity as they can release lipid mediators, oxidative products, extracellular DNA traps and Charcot‐Leyden crystals (CLC) [7].

Despite various treatment methods for CRSwNP including saline irrigations, intranasal and oral corticosteroids, antibiotics, and surgical approaches, the disease often requires lifelong treatment [8, 9]. Especially patients having a type 2‐high inflammation have major risks for recurrence and asthma comorbidity [10, 11, 12]. For these patients, recent advances emerged by the development of biologics or monoclonal antibodies, targeting specific cytokines for the survival and trafficking of eosinophils, but also interfere with type 2 inflammatory pathways, independent of eosinophils. Biologics are widely used to treat severe uncontrolled eosinophilic asthma but are only recently approved for CRSwNP treatment. Rendering knowledge on their mechanisms in this disease is scarce.

Mepolizumab (anti‐IL‐5) interferes with eosinophil‐mediated inflammation. Clinical studies showed a significant reduction in need for surgery and disease severity, including nasal polyp size, nasal congestion, obstruction, and facial pain, next to reducing the blood eosinophil count [13, 14, 15]. Benralizumab, targeting IL‐5 receptor alpha, also reduces nasal polyp scores and blood eosinophils but does not significantly improve Sinonasal‐Outcome‐Test‐22 scores [16]. On the other hand, studies with omalizumab (anti‐IgE) showed that treatment ameliorated clinical symptoms of CRSwNP, reduced nasal polyp size, but had only limited effects on blood eosinophil levels [17]. Remarkable, omalizumab is effective in CRSwNP patients with comorbid asthma, independent of the presence of allergy and blood eosinophils [18]. Dupilumab, designed against the alpha subunit of the IL‐4 receptor, inhibits IL‐4 and IL‐13 signaling. The antibody leads to both a significant reduction in nasal polyp size and tissue eosinophils, and improvement of patient symptoms [19, 20]. However, the exact mechanisms by which biologicals exert effects on the local polyp eosinophilic inflammation must be further investigated.

The effectiveness of the biological treatment is between 50% and 75% [5]. Compared to standard of care, the treatments improve health‐related quality of life. Despite the induced changes in total nasal polyp score, the nasal polyps are rarely completely eradicated [21]. In case the residual polyps cause unresolved nasal problems, sinus surgery may still be indicated. Biological treatments do not work for all patients, and prediction of effects in an individual patient by blood or tissue eosinophils fails [22, 23]. Recently, we discovered that type 2‐high patients treated with omalizumab showed an increased neutrophil infiltration [24]. We therefore were interested in clarifying the role of neutrophils in CRSwNP, acknowledging that neutrophils have been linked to a more severe disease with reduced responsiveness to oral corticosteroids in both CRSwNP and asthma [25].

The aim of this study was to investigate how different biologics modulate type 2 airway inflammation and to understand the response to these treatments in terms of changes in eosinophil, but also neutrophil granulocytes.

## 2 Methods

### 2.1 Patient Material

Nasal polyp samples were collected from 54 different type 2‐high (IL‐5 > 12.98 pg/g tissue [6]) CRSwNP patients, at Ghent University Hospital (Belgium), after written informed consent, approved by local ethical committees. Of these, 41 patients were under biological treatment (benralizumab, mepolizumab, omalizumab, or dupilumab) for at least 16 weeks at the moment of biopsy, whereas 13 patients were never treated with a biological. Biopsies were collected by endoscopy, and all samples were included in the study based on availability and eligibility. Exclusion criteria were immunotherapy or the use of systemic corticosteroids or antibiotics within 6 weeks prior to the intervention. Diagnosis was based on patient history, skin prick tests, clinical examination, nasal endoscopy and/or computed tomography. Detailed demographic information and clinical baseline characteristics are provided in Supporting Information S1: Table S1. Asthma prevalence varied significantly across groups, however, no individual pairwise comparison remained significant after correction for multiple comparisons. Exploratory Spearman correlations found no association between tissue MPO or ECP levels and asthma status (data not shown), suggesting asthma does not confound our inflammatory markers. Comparisons were made between treated and untreated (CRSwNP) patients groups, having similar baseline characteristics (see Supporting Information S1: Table S1), and/or within the same patient before and after biologic therapy. The type of comparison (inter‐individual or paired intra‐individual) is indicated in each figure.

### 2.2 Immunohistochemistry

Tissue samples were fixed in 10% buffered formalin for 24 h at room temperature, embedded in paraffin, and cut into 4 μm sections. After paraffin removal with xylene, the sections were rehydrated in gradually decreasing steps of ethanol in water. This is followed, for the eosinophil staining, by a heat‐mediated antigen retrieval step using citrate buffer. Next, a blocking step in 7.5% bovine serum albumin to prevent nonspecific binding and staining with primary antibodies anti‐elastase (Agilent Technologies, Santa Clara, USA), anti‐galectin‐10 (R&D Systems, Bio‐Techne, Abingdon, UK). To ensure staining specificity, all primary antibodies were validated and matched with isotype controls. Following, the alkaline phosphatase–linked secondary antibody kit (Agilent Technologies) was used conform the manufacturer's instructions, and sections were counterstained with hematoxylin. The sections were scanned under consistent imaging settings with the Nikon ECLIPSE Ni‐E microscope (Nikon, Tokyo, Japan). Image analysis was performed using the NIS Elements imaging software (Nikon). The total tissue area was automatically identified, and positive staining was quantified via standardized thresholding. Pixel‐level segmentation determined the proportion of positively stained cells relative to the total area.

### 2.3 Protein Measurements

Tissue samples were snap‐frozen after surgical collection and stored at −80°C. For extraction, samples were mechanically disrupted and homogenized using the TissueLyser LT (Qiagen, Germantown, USA). Homogenates were 11 times diluted with sterile 0.9% NaCl solution (Braun, Melsungen, Germany), supplemented with a protease inhibitor cocktail (Roche Diagnostics, Basel, Switzerland). Next, the supernatant was separated from the suspension by centrifugation. Multiplex cytokine and chemokine levels were measured using the Luminex Performance Assays (R&D Systems), and analyzed on the Bio‐Plex 200 system (Bio‐Rad, California, USA), following the manufacturer's protocols. Internal controls and standard curves were included in each plate to assess linearity, sensitivity, and reproducibility. Total IgE and ECP levels were determined with the ImmunoCAP system (Thermo Fisher Diagnostics, Merelbeke, Belgium). Values below the detection limit were given a substitution value equaling half the detection limit.

### 2.4 Bulk RNA Sequencing

Total RNA was isolated from snap‐frozen nasal tissue using the RNeasy Mini Kit (Qiagen), including on‐column DNase digestion. RNA concentration was measured and integrity was verified with dual‐platform assessment: Nanodrop spectrophotometry (Life Technologies, Grand Island, NY, USA) was used to measure purity (A260/280 and A260/230 ratios), and the Fragment Analyzer System (Agilent Technologies) confirmed RNA integrity via the RNA Quality Number. A total of 500 ng of RNA was used for library construction with the Illumina stranded mRNA ligation kit and RNA UDI adapters (Illumina, San Diego, CA, USA) according to the protocol for long inserts. Libraries were amplified via enrichment PCR (14 cycles) and quantified by qPCR, according to Illumina's “Sequencing Library qPCR Quantification protocol guide.” Size distribution and integrity of libraries were checked with a high‐sensitivity DNA chip (Agilent Technologies), and equimolar pooling ensured balanced representation. Pooled libraries were additionally purified with E‐Gel EX 2% agarose gel (Life Technologies). Sequencing was performed on an Illumina NovaSeq 6000 S4 lane with 5% PhiX Sequencing Control library, generating 150 bp paired‐end reads. Post‐sequencing, the reads were trimmed to remove adapter contamination and low‐quality bases. Next, the reads were mapped against the *Homo sapiens* GRCh38 reference genome using STAR aligner software [26]. RSEM‐software was used to generate gene and transcript‐level counts [27]. Differentially expressed genes were identified using DESeq2 [28]. Genes were considered differentially expressed when the Benjamini‐Hochberg‐adjusted *p*‐value < 0 0.05 and log2 fold change > 1 or < −1. Heatmaps were generated with pheatmap, based on VST‐transformed counts from DESeq2 and *Z*‐scale transformation. Volcano plots were computed using ggplot [29]. Pathway analysis was performed using clusterProfiler for gene set enrichment analysis of Gene Ontology (GO) gene sets [30].

### 2.5 Single‐Cell RNA Sequencing

Nasal polyp single‐cell data was used from the database previously published by our lab [31]. Shortly, a single cell suspension was made of fresh nasal polyp tissue, using a gentleMACS Dissociator (Miltenyi Biotec, Leiden, The Netherlands). One million cells were stained with TotalSeq‐C Human Universal Cocktail antibodies (BioLegend, San Diego, USA), and 125,000 live cells were sorted. Cells were loaded on the droplet‐based chromium microfluidic system using Next GEM Single Cell 5′ Gel Bead Kit v2 (10× Genomics, Leiden, The Netherlands). Gene expression and feature barcodes libraries were prepared and subjected to 13 and 8 amplification cycles, respectively. Libraries were sequenced with HiSeq 3000 System (Illumina) to achieve minimum 25,000 paired‐end reads per cell for gene expression, and 5000 paired‐end reads per cell for cell surface protein expression. Data from samples were integrated and analyzed with harmony and Seurat [32, 33]. The sequencing data were processed: First Cell Ranger was used to generate gene expression and surface protein data. Next, quality control was conducted with Scater and scDbIFinder, low‐quality cells and doublets were removed [34, 35]. Background signals from empty droplets were removed with SoupX [36]. Cells were clustered with the Louvain algorithm based on gene expression matrices and annotated according to the expression of canonical cell‐type markers. Graphs were computed using Seurat and ggplot [29].

### 2.6 Statistical Analysis

Statistical data analysis was performed with Prism 9 (GraphPad, California, USA). Normality was tested with the Shapiro–Wilk test. The unpaired non‐parametric Mann–Whitney *U* two‐tailed test was used to compare two non‐normal distributed groups. A two‐sided Fishers' exact test was utilized to compare categorical outcomes. To compare multiple unpaired groups, the non‐parametric Kruskal–Wallis test was utilized with a Dunn's test for multiple comparisons. The comparison between two paired groups was conducted with the non‐parametric Wilcoxon matched pairs signed rank test. *p*‐values ≤ 0.05 were considered statistically significant, with following significance levels *p ≤ 0.05; **p ≤ 0.01; ***p ≤ 0.001; ****p ≤ 0.0001.

## 3 Results

### 3.1 Distribution of Eosinophils and Neutrophils in Nasal Polyp Tissue, Before and After Treatment With Monoclonal Antibodies

**FIGURE 1:**
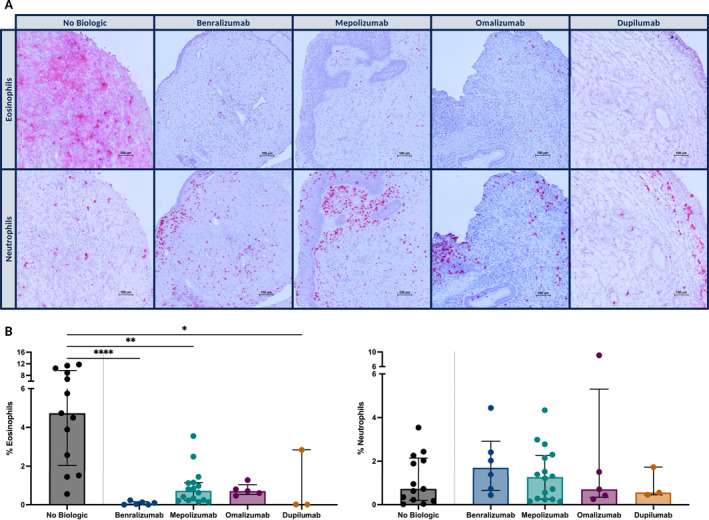
Immunohistochemical staining of nasal polyp tissue for eosinophils and neutrophils. (A) Representative sections of nasal polyp tissue stained with galectin‐10 (top row) for eosinophils with elastase (bottom row) for neutrophils. Columns show samples from untreated patients column (1), and those treated with benralizumab (2), mepolizumab (3), omalizumab (4), or dupilumab (5). (B) Semi‐quantitative analysis of eosinophils (left) and neutrophils (right), expressed as percent positive signal relative to the total tissue area, of patients without treatment (13 patients) and patients after treatment with either benralizumab (6 patients), mepolizumab (16 patients), omalizumab (5 patients) or dupilumab (3 patients) (B). Statistical comparisons were performed on the inter‐individual comparison using the Kruskal–Wallis test with a Dunn's test for multiple comparisons (*p ≤ 0.05, **p ≤ 0.01 and ****p ≤ 0.0001).

Prior to treatment, the patients had similar characteristics as a group of untreated CRSwNP patients. There was no significant difference between median age, gender, allergy and asthma status, amount of revision surgeries, and smoking status (Supporting Information S1: Table S1). Also, nasal polyp scoring, Lund‐Mackay scores and the general nasal/sinus symptom scores were not significantly different between groups. Immunohistochemistry staining for eosinophils (galectin‐10) and neutrophils (elastase) was performed on nasal polyp tissue of a selection of patients. Nasal tissue of type 2‐high CRSwNP patients, without biological treatment (*n* = 13) showed large amounts of infiltrating eosinophils, while only a small number of neutrophils were present (Figure 1A, first column). After treatment with either benralizumab (*n* = 6), mepolizumab (*n* = 16), omalizumab (*n* = 5), or dupilumab (*n* = 3), we found that these eosinophils were largely reduced in the nasal tissue (Figure 1A, first row, and 1B, left). Neutrophils stayed or seemed slightly increased in the tissue by the biologics, mainly situated in and under the epithelial cell layer of the tissue (Figure 1A, second row, and 1B, right).

### 3.2 Eosinophil and Neutrophil Secretory Proteins Were Differential After Treatment With Biologics

**FIGURE 2:**
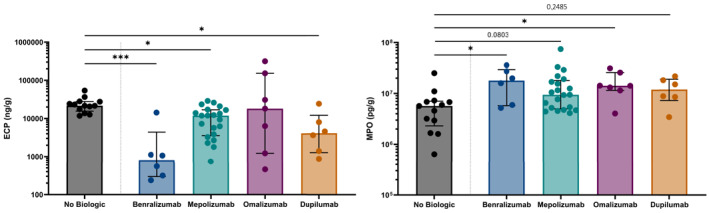
Protein levels of eosinophilic cationic protein (ECP) and myeloperoxidase (MPO) in nasal polyp tissue. Protein concentrations of ECP (left) and MPO (right) were measured on nasal polyp tissue from untreated patients (13 patients) and patients treated with benralizumab (6 patients), mepolizumab (21 patients), omalizumab (7 patients) or dupilumab (6 patients). Assays were performed using the Luminex Performance Assays and ImmunoCAP systems. Statistical comparisons were made on the inter‐individual comparison using the Kruskal–Wallis test with a Dunn's test for multiple comparisons (*p ≤ 0.05 and ***p ≤ 0.001).

Previous results were confirmed through protein measurements of ECP, from eosinophil granules, and myeloperoxidase (MPO), from neutrophil granules, in polyp tissue. ECP was significantly downregulated by benralizumab (*n* = 6), mepolizumab (*n* = 21), and dupilumab (*n* = 6) treatments, but not by omalizumab (*n* = 7), compared to patients who didn't receive those treatments (*n* = 13) (Figure 2, left). This was in accordance with previous eosinophil stainings (Figure 1). When comparing tissues from the same patient before and after treatment (paired intra‐individual comparison), ECP was downregulated after treatment with mepolizumab (*n* = 5) and dupilumab (*n* = 6) (see Supporting Information S1: Figure S1, first row, left). More specifically, it was decreased in each patient by mepolizumab and dupilumab, but mixed responses with even high increases of ECP could be observed after omalizumab (*n* = 4) (see Supporting Information S1: Figure S1, second row). Compared to untreated patients (inter‐individual comparison), MPO was significantly upregulated by benralizumab, omalizumab and as trend by mepolizumab (Figure 2, right). When comparing intra‐individual, MPO was only downregulated after dupilumab (see Supporting Information S1: Figure S1, first row, right). Specifically, MPO was increased in four of five cases by mepolizumab and by omalizumab, while dupilumab led to a reduction of MPO in four of the six cases (see Supporting Information S1: Figure S1, third row).

The inter‐individual comparison also highlighted that benralizumab significantly increased IP‐10, FGF‐β, IL‐10 and GM‐CSF (decreasing order, see Supporting Information S1: Table S2). Of note, mepolizumab led to a significant upregulation of IL1RA (2.8×) and granzyme B (3.6×). Omalizumab resulted in a significant upregulation of IL‐1β, granzyme B, IL‐2, IL‐8, G‐CSF, TNF‐α, GM‐CSF, and Flt‐3L (decreasing order) inter‐individually. Dupilumab led to a significant twofold increase of IL‐1RA, and a significant downregulation of PDGF‐AB/BB, IgE, RANTES, IL‐3, eotaxin, PD‐L1/B7‐H1, PDGF‐AA, MIP‐1β, EGF, and GRO‐β (decreasing order).

### 3.3 Divergent Expression of Genes for Eosinophils and Neutrophils After Treatment With Biological Drugs

**FIGURE 3:**
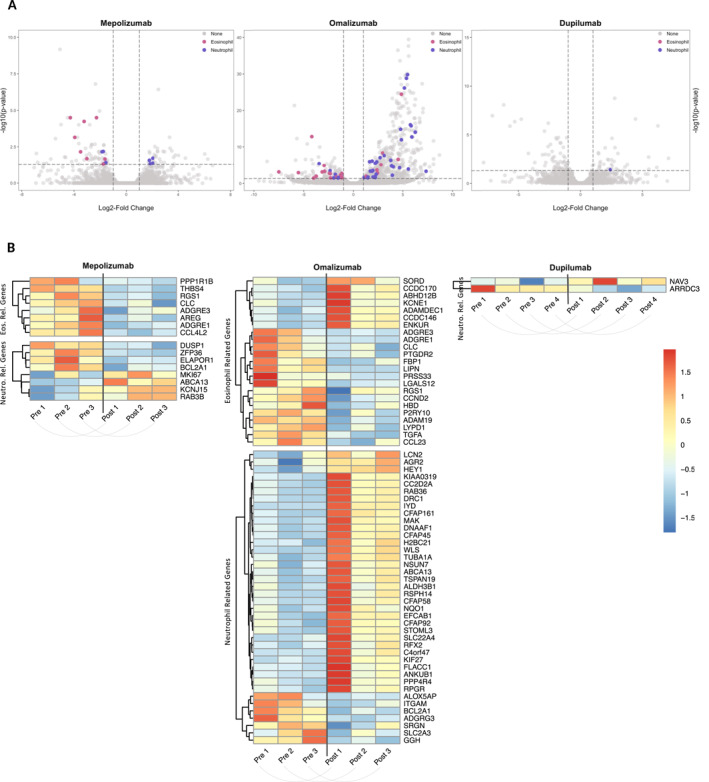
Gene expression profiles following biological treatment in CRSwNP patients. Bulk RNA sequencing was performed on paired nasal polyp samples from patients before and after treatment with mepolizumab (3 patients, 6 samples), omalizumab (3 patients, 6 samples) and dupilumab (4 patients, 8 samples), resulting in a paired intra‐individual comparison. (A) Volcano plots display the differentially expressed genes, determined using DESeq2 (adjusted *p* < 0.05; absolute log2 fold change > 1), with eosinophil‐related genes in pink and neutrophil‐related genes in blue. (B) Heatmaps depict *z*‐score‐scaled expression of the differentially expressed eosinophil‐related (Eos. Rel. Genes) and neutrophil‐related (Neutro. Rel. Genes) across the individual patients.

From 10 patients, nasal polyp samples were available before and after treatment with biologics, on which we performed bulk RNA sequencing. Differential expressed genes (DEGs) were determined using DESeq2, resulting in 105 DEGs for mepolizumab (*n* = 3), 664 for omalizumab (*n* = 3) and 107 for dupilumab (*n* = 4). Genes were compared with either characteristic eosinophil genes, defined by the eosinophil dataset from Human Protein Atlas and published characteristic airway eosinophil genes [37], or genes for their neutrophil counterparts, defined by the neutrophil dataset from Human Protein Atlas and genes from neutrophil granules [38] (Figure 3A). After mepolizumab treatment, genes related to eosinophils were all downregulated compared to before treatment (Figure 3B, left). Most of these genes were defined within the innate immune response cluster (i.e., THBS4, RGS1, ADGRE3). Four neutrophil‐related genes were downregulated, also mostly clustered within the innate immune response (ZFP36, ELAPOR1, and BCL2A1), while four others were upregulated (Figure 3B, left). Omalizumab treatment led to a decrease of the majority of DEGs for eosinophils, mostly clustered within the innate immune response (i.e., ADGRE3, ADGRE1, FBP1) (Figure 3B, middle). In accordance with MPO measurements, most of the neutrophil genes were upregulated after omalizumab treatment (Figure 3B, middle), belonging to different clusters such as cellular senescence (i.e., HEY1, DRC1, DNAAF1), innate immune response (i.e., KIAA0319, RAB36, CFAP161), chromatin organization (i.e., CC2D2A, IYD, FLACC1), inflammatory response (i.e., WLS, TUBA1A), or mixed function (i.e., CFAP45, TSPAN19, RSPH14). Some neutrophil‐associated genes were downregulated, mostly encoding for the granule proteins of neutrophils (ITGAM, SLC2A3, GGH). After dupilumab, only two neutrophil genes were differentially expressed (Figure 3B, right).

### 3.4 Bulk RNA Sequencing Shows Up‐ and Downregulation of Markers for Neutrophil and Eosinophil Chemoattraction

**FIGURE 4:**
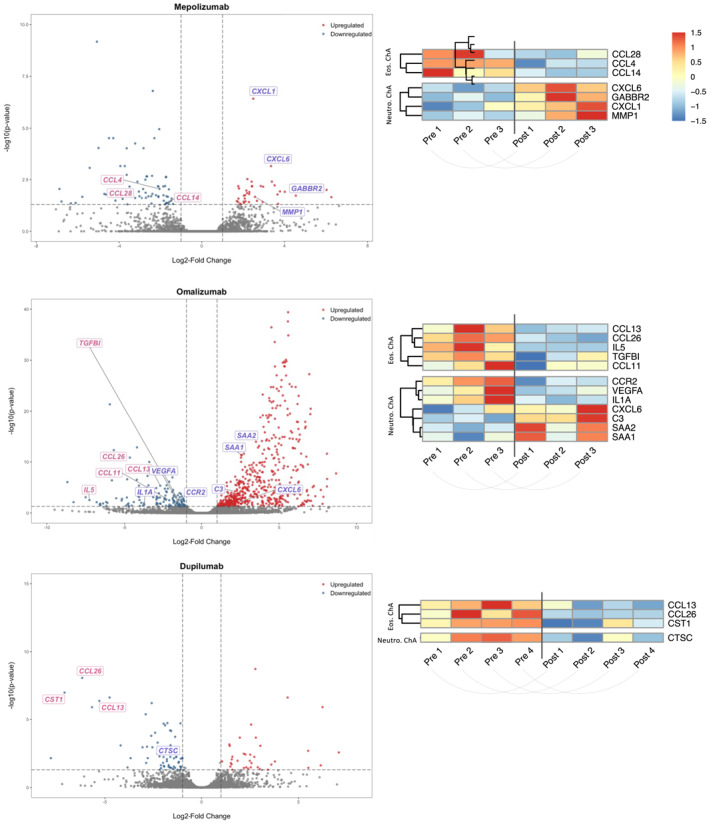
Expression of chemoattractant genes for neutrophils and eosinophils in CRSwNP tissue. Bulk RNA sequencing was performed on paired nasal polyp samples from patients before and after treatment with mepolizumab (3 patients, 6 samples), omalizumab (3 patients, 6 samples) and dupilumab (4 patients, 8 samples), resulting in a paired intra‐individual comparison. Volcano plots show the differentially expressed genes, determined using DESeq2 (adjusted *p* < 0.05; absolute log2 fold change > 1), of eosinophil (pink) and neutrophil (blue) chemoattractant genes. Heatmaps display *z*‐score‐scaled expression of these genes, separated into eosinophil‐associated (Eos. ChA) and neutrophil‐associated (Neutro. ChA) subsets.

The DEGs were also compared to genes related to eosinophil and neutrophil chemotaxis. After mepolizumab treatment, eosinophil chemokines CCL4, CCL14, and CCL28 diminished, while neutrophil‐chemotactic genes showed an upregulation, such as CXCL1, CXCL6, MMP1, and GABBR2 (Figure 4, top). Omalizumab treatment resulted in similar downregulation of eosinophilic chemokine genes, mainly concerning the eotaxins (CCL11, CCL26), CCL13, IL5, and TGFBI (Figure 4, middle). The neutrophilic chemotactic genes IL1A and VEGFA were downregulated, however, most neutrophil chemokines (C3, SAA1, SAA2, and CXCL6) were rather upregulated (Figure 4, middle). In contrast to mepolizumab and omalizumab, dupilumab treatment did not lead to an upregulation of neutrophil chemoattractant genes, but even a downregulation of CTSC (Figure 4, bottom). This was observed in combination with a downregulation of the eosinophil chemokines CST1, CCL13, and CCL26 (Figure 4, bottom).

### 3.5 Single‐Cell RNA Sequencing Reveals the Cellular Source of the Chemoattractant Genes

**FIGURE 5:**
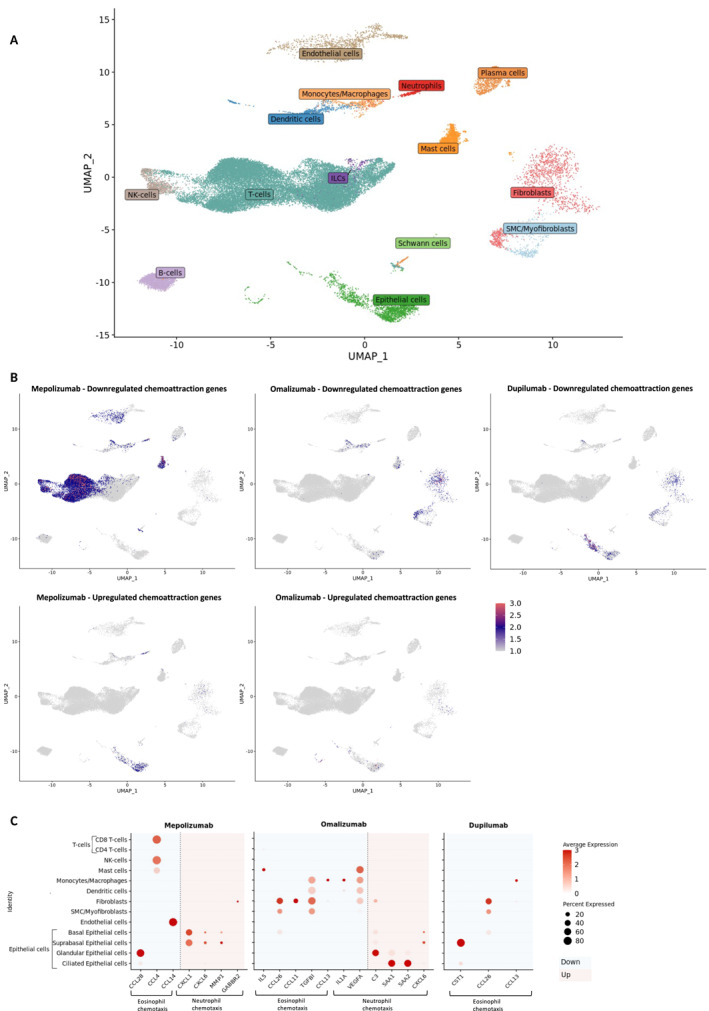
Single‐cell expression of neutrophil and eosinophil chemoattractant genes in nasal polyps. (A) UMAP plot display 14 cell clusters identified in scRNA‐seq of untreated nasal polyp tissue (4 patients). (B) Combined feature plots illustrate spatial expression of up‐ and downregulated neutrophil and eosinophil chemoattractant genes, previously identified by bulk RNA‐sequencing (paired intra‐individual comparison). (C) Dot plots summarize cell‐type expression patterns of the chemoattractant genes: dot size represents the percentage of expression; color intensity reflects the average expression. Downregulated genes are shown in blue, upregulated genes in red.

Single‐cell RNA sequencing identified 14 major cell types in nasal polyp tissue (*n* = 4, Figure 5A). Unfortunately, eosinophils could not be detected during the sequencing as they were hard to capture using the 10× Genomics chromium platform. In this dataset, cellular localization was investigated by plotting the combination of previous identified chemokines as a group. The eosinophil chemokines, downregulated after mepolizumab treatment, were derived from epithelial and endothelial cells, mast cells, NK‐, and T‐cells (Figure 5B, left). Specifically, expression of CCL28 was mainly derived from glandular epithelium, CCL4 from CD8^+^ T‐cells and NK‐cells, and CCL14 from endothelial cells (Figure 5C, left). Upregulated neutrophil chemoattractant genes were mainly derived from epithelial cells (Figure 5B, left), namely (supra)basal epithelial cells and fibroblasts (Figure 5C, left). GO enrichment analysis for DEGs before and after mepolizumab treatment, found with bulk RNA sequencing, showed that pathways for muscle development were downregulated (Supporting Information S1: Figure S2, left). The downregulated chemoattractant genes after omalizumab could be found within the (myo)fibroblast, monocyte/macrophage, and mast cell clusters (Figure 5B, middle). This was consistent with pathway analysis, based on the bulk RNA sequencing, showing that after omalizumab treatment, monocyte chemotaxis was suppressed (Supporting Information S1: Figure S2, middle). The two downregulated neutrophil chemoattractant genes (IL1A and VEGFA) could also be found within these clusters (Figure 5C, middle), while the upregulated neutrophil chemoattractant genes were mainly situated in the epithelial cluster, especially from glandular and ciliated cells (Figure 5C, middle). Enrichment analysis revealed that pathways related to cilia movement and organization were activated (Supporting Information S1: Figure S2, middle). In contrast to mepolizumab treatment, downregulated chemoattractant genes after dupilumab were in (supra)basal epithelial and (myo)fibroblast clusters (Figure 5B,C, right). After dupilumab, many chemotactic pathways, such as monocyte, neutrophil, and lymphocyte chemotaxis, were suppressed (Supporting Information S1: Figure S2, right).

### 3.6 The Localization of Receptors of Biologics in Nasal Polyp Tissue

**FIGURE 6:**
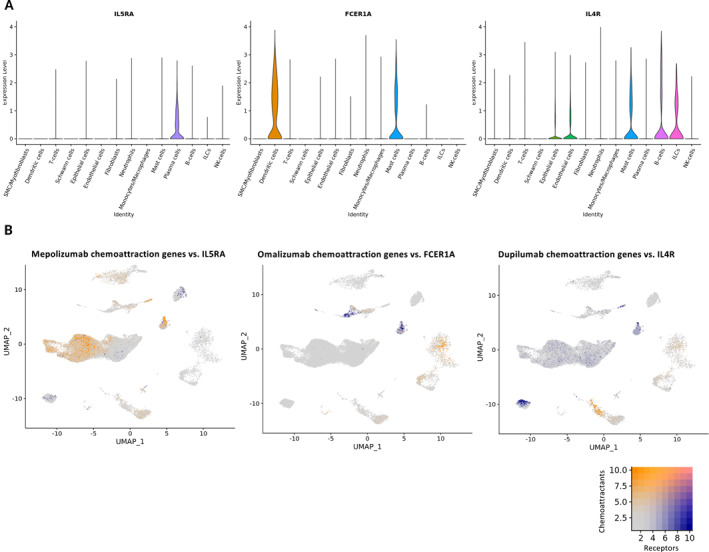
Cellular distribution of biologic receptor targets in nasal polyp tissue. (A) Violin plots show cell‐type‐specific expression of IL5RA, FCER1A or IL4R in nasal polyp tissue (4 patients), using Seurat. (B) Feature plots showing co‐expression of eosinophil and neutrophil chemoattractant genes (orange) with the associated receptor targets (blue) across treatment arms: mepolizumab (left), omalizumab (middle) or dupilumab (right).

In the single‐cell dataset, we checked the expression of receptors, involved in the direct pathways of the biologics. The IL‐5 receptor, targeted by benralizumab and indirectly by mepolizumab, could be mainly found on plasma cells (Figure 6A, left). FCER1A, the receptor gene targeted in the omalizumab pathway, was expressed on dendritic cells and mast cells (Figure 6A, middle). The common IL‐4 and IL‐13 receptor, targeted by dupilumab, was found on epithelial cells, endothelial cells, mast cells, B‐cells and ILCs (Figure 6A, right). However, when the cellular localization of the previously combined chemoattractant genes for eosinophils and neutrophils (blue) was compared to the cells, on which the receptors for the biological targets were present (orange), there was no overlap observed of these cells for any of the treatment methods (Figure 6B).

## 4 Discussion

CRSwNP is mostly a type 2‐high prone disease, characterized by eosinophil tissue infiltration. Current treatment methods, such as corticosteroids or biologics, target the type 2 inflammation. Our data showed that biologics benralizumab, mepolizumab, omalizumab, and dupilumab exert distinct effects on local immune pathways. All resulted in a downregulation of proteins and genes related to the eosinophilic inflammation and chemoattraction. However, only benralizumab, mepolizumab, and omalizumab showed a simultaneous upregulation of neutrophilic markers, possibly indicating a shift from eosinophilic to neutrophilic inflammation in these patients. Chemoattractant genes for neutrophils were mainly derived from epithelial cells. These effects were secondary as they didn't overlap with cells having receptors for the biologics.

Despite type 2‐high CRSwNP being predominantly characterized by eosinophils, data suggest that targeting eosinophils for depletion alone is not sufficient as treatment method. Benralizumab blocks the IL‐5 receptor, present on eosinophils and plasma cells, and shows the highest decrease in eosinophils and ECP. This was in agreement with previous studies, showing reduced blood eosinophil numbers and sera ECP after benralizumab in asthma [39]. However, in comparison with mepolizumab, omalizumab and dupilumab, benralizumab was shown to be the least effective for the treatment of CRSwNP in terms of reducing nasal congestion severity and outcome scores [40]. Studies using dexpramipexole, significantly lowering eosinophil counts in blood and tissue, didn't improve nasal symptoms nor the nasal polyp score [41]. It suggests that, next to eosinophils, other cells have significant roles in nasal polyposis. We observed that in the benralizumab and omalizumab group, there was a significant increase of the neutrophil protein MPO after treatment. Mepolizumab treatment also showed an upward trend in MPO levels, although this did not reach statistical significance. This was surprising, as a more prominent neutrophilic inflammation in CRSwNP patients was mostly described in the Asian population [42, 43]. In both asthma and CRSwNP, a severe neutrophilic inflammation is linked to reduced responsiveness to corticosteroids [25, 44]. There are several trials to target neutrophils in asthma, however, no successful target has been identified yet [45]. Nonetheless, new biological strategies target epithelial‐derived alarmins such as IL‐33 (etokimab, itepekimab) and thymic stromal lymphopoietin (tezepelumab) in CRSwNP [46]. In several asthma trials, it was shown that tezepelumab significantly reduces exacerbations, independent of baseline blood eosinophil counts, and could be a promising treatment option [47]. In line with our tissue‐molecular findings, the first phase 3 data for TLSP inhibition in CRSwNP have just been reported. In the WAYPOINT trial, Tezepelumab produced reductions in the nasal polyp score and the nasal congestion score, improved the loss‐of‐smell score, and also nearly eliminated the need for subsequent polyp surgery compared to placebo [48].

Although effects of these biologics are similar, they have distinct working mechanisms: Mepolizumab prevents IL‐5 to bind the IL‐5Rα subunit on eosinophils, plasma cells, mast cells and ciliated epithelial cells [49]. Omalizumab reduces free IgE in circulation and leads to reduced numbers of high‐affinity IgE receptors on basophils, mast cells and dendritic cells [50]. Dupilumab targets the IL‐4Rα subunit on various cells to inhibit IL‐4/IL‐13 signaling, crucial for recruitment of immune cells, as eosinophils and Th2‐cells, to the local tissue. All tested biologics led to a decrease in eosinophil chemotaxis genes. Remarkably, most of these genes were distinct within the treatment groups, suggesting unique pathways/regulation. Unlike the others, dupilumab did not lead to an increase of neutrophil chemoattractant genes. Treatment duration varied across the groups, with dupilumab administered for a shorter period, relative to the other biologicals. Despite this difference, dupilumab already induced significant molecular changes. We believe that the duration of the treatment is unlikely to have significantly impacted the results regarding neutrophil influx, supported by the lack of correlation between treatment duration and MPO‐levels (*p* = 0.8029, *r* = −0.04). Additionally, clinical experience and previous studies suggest that patients treated with dupilumab often exhibit a rapid therapeutic response, with achievement of clinically meaningful responses by week 16 which is maintained at week 52, which may further mitigate the impact of shorter treatment duration [51, 52]. Noteworthy, it was shown that dupilumab has the greatest improvement in outcomes for CRSwNP [21].

Effector cells, leading to chemotaxis of eosinophils and neutrophils in nasal polyps were also distinct depending on the drug. The downregulated eosinophil chemokine genes by dupilumab treatment, as observed in the bulk RNA sequencing, is associated with the (supra)basal epithelial and (myo)fibroblast clusters in our single‐cell reference dataset. On the contrary, the latter clusters were the cell types leading to upregulated neutrophil chemoattractant genes by mepolizumab. Additionally, after omalizumab treatment, these neutrophil attractant genes were all mainly associated with the epithelial cluster in the reference dataset, especially derived from glandular and ciliated epithelial cells. This indicates that epithelial cell changes could trigger neutrophils to infiltrate the tissue. Indeed, we know that the epithelial barrier is affected in CRSwNP, especially in type 2‐high patients [24]. In the epithelial layer, there is a loss of ciliated cells and decreased tight junction proteins, leading to lower trans‐tissue resistance [53, 54]. Our data would suggest that treatment with different biologics induces tissue repairs through distinct modes of actions. IL‐4 could decrease tissue resistance even more, suggesting that dupilumab could help restore the epithelial resistance [54]. Remarkably, here we observed that omalizumab led to increased ciliated cell‐related genes, which was in turn the cell type inducing neutrophil chemoattractant genes. Another possible explanation is that despite disappearance of eosinophils, eosinophil products stay and exert effects. During eosinophil death, CLCs are released, which can trigger the epithelium to recruit neutrophils in CRSwNP [55, 56]. In turn, these neutrophils can produce oncostatin M that can disrupt the epithelial barrier and lead to type 2‐inflammation [57]. Additionally, the observed changes are secondary effects, as the cellular localization of the chemoattractant genes was different than cells having receptors for the biological targets.

This study has limitations: First, due to reliance on archived frozen tissue, advanced techniques such as single cell sequencing or spatial transcriptomics, were not feasible. While bulk RNA sequencing provided robust and informative transcriptional insights, cellular resolution was restricted. Follow‐up studies will be necessary to dissect the cell‐type‐specific regulatory pathways underpinning the biologic responses. Secondly, the sample sizes, particularly for certain treatment groups, were limited, and matched clinical follow‐up data, clinical markers (e.g., N‐ERD status, environmental exposures to allergens), or systemic biomarker data (e.g., blood eosinophils, serum IgE) were not consistently available. These factors limited the ability to perform prediction modeling or correlation analyses between biomarkers and treatment outcomes. These constraints highlight the need for prospective, longitudinal studies with standardized clinical and biomarker assessments to better explore causality and the predictive utility of tissue‐based markers.

Despite these limitations, our study, integrating immunohistochemistry, protein profiling and transcriptomics, gives novel insights to the distinct immunologic effects of four biologics in CRSwNP and provides initial evidence of treatment‐associated molecular changes in nasal tissue, that warrant prospective validation in randomized studies. We show treatment‐induced suppression of eosinophil‐associated inflammation and unique patterns of neutrophil chemoattractant gene expression. In a clinical context, tissue‐level profiling might serve as a complementary approach to monitor therapeutic response. These findings might also lay the groundwork for future stratification strategies and personalized treatment planning.

To conclude, monoclonal antibody treatment has plenty effects apart from their primary inhibitory goals. The biologics lead to reduced eosinophilic inflammation. However, benralizumab, mepolizumab, and omalizumab lead to a simultaneous upregulation of neutrophilic markers in the tissue. The possible chemoattraction of neutrophils could be due to secondary effects and associated with the epithelial barrier.

## Author Contributions

S.V.N., N.Z., S.V., P.G. and C.B. conceptualized and designed the methodology. S.V.N., G.H. and N.D.R. performed the experiments. S.V.N. and Z.X. analyzed the data. M.B. and E.V. investigated clinical data. F.V.N. provided resources. S.V.N. wrote the original draft. S.V.N., E.V., S.V. and P.G. revised the manuscript.

## Conflicts of Interest

Claus Bachert participated in advisory boards and received speaker fees from AstraZeneca, GlaxoSmithKline, Novartis, Regeneron, and Sanofi Genzyme. Philippe Gevaert participated in advisory boards and received speaker fees from ALK‐Abelló, Argenx, AstraZeneca, Genentech, GSK, Insmed, Lilly, Novartis, Regeneron, Roche, Sanofi Genzyme, and Stallergenes‐Greer.

## Supporting information


Supporting Information S1

## Data Availability

The data that support the findings of this study are available from the corresponding author upon reasonable request.
