# Supplementary material for: Distinct Effects of Biological Treatments on Eosinophils and Neutrophils in Chronic Rhinosinusitis With Nasal Polyp Patients
Source: Clin Transl Allergy. 2025 Nov 27;15(12):e70117. doi: 10.1002/clt2.70117 (PMC12660500; doi:10.1002/clt2.70117)
Supplement: Supplementary file 1 — Supporting Information S1 [file CLT2-15-e70117-s001.pptx]

## Slide 1
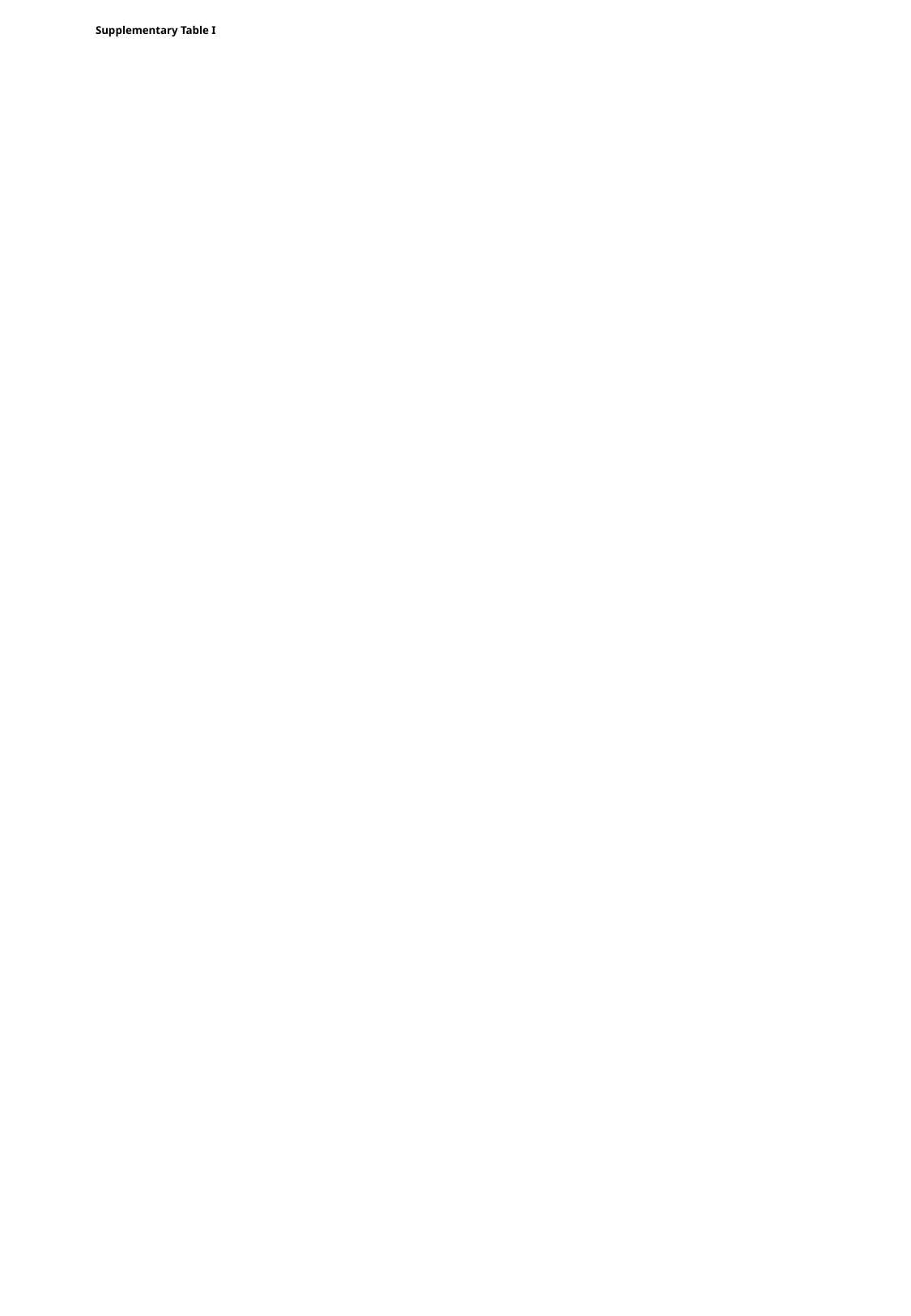

Supplementary Table I

## Slide 2
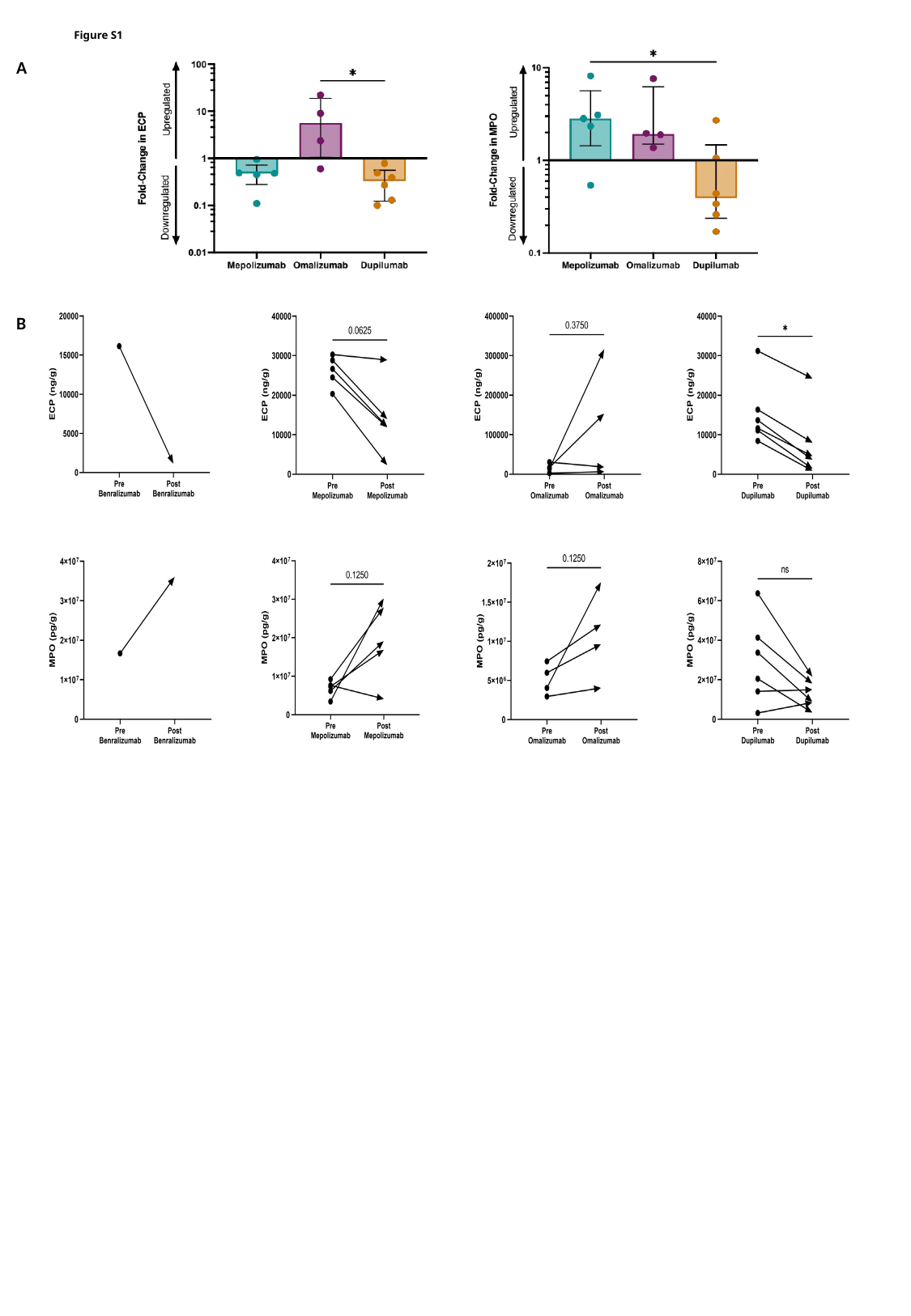

Figure S1
A
B

## Slide 3
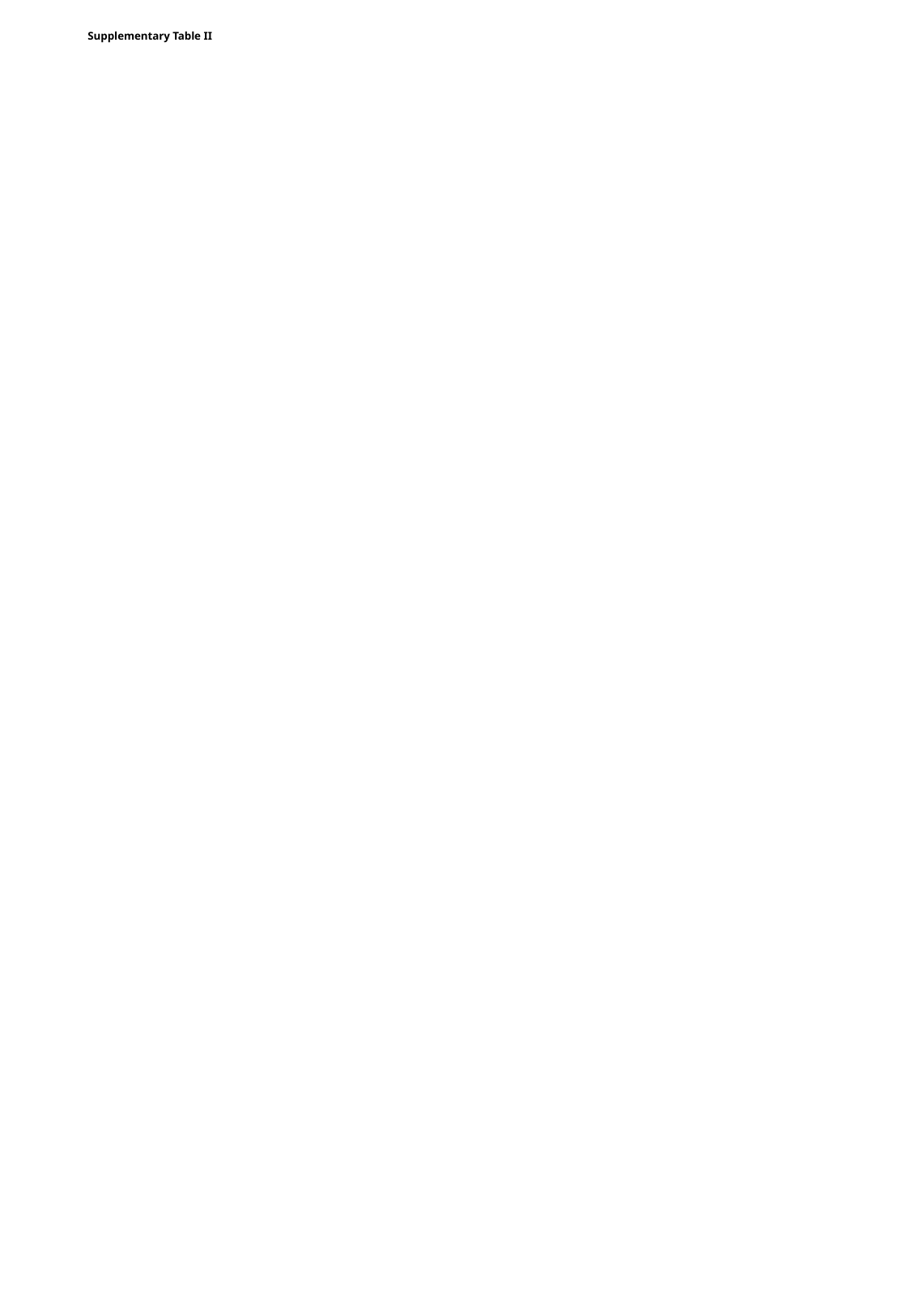

Supplementary Table II

## Slide 4
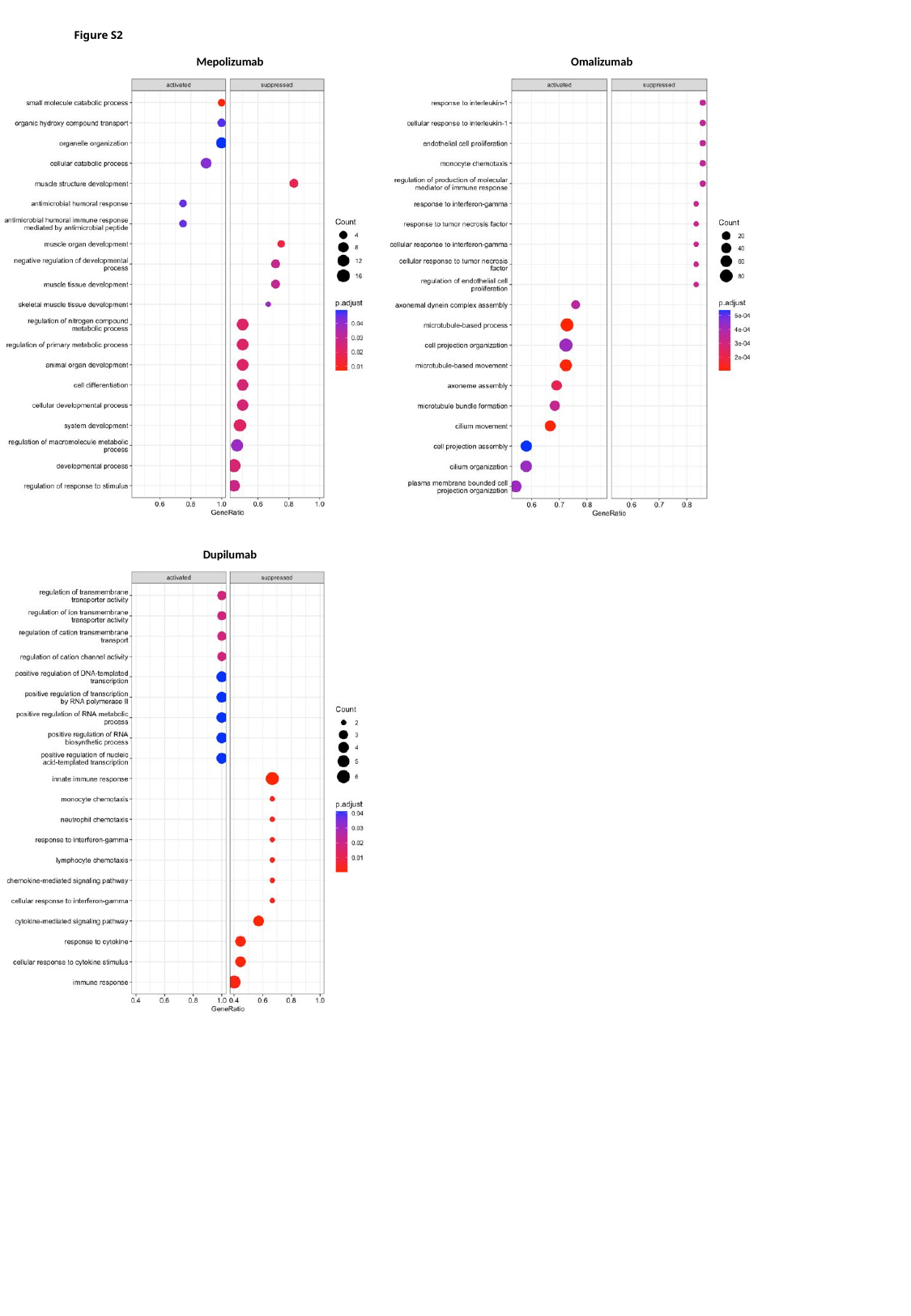

Figure S2
Omalizumab
Mepolizumab
Dupilumab

## Slide 5
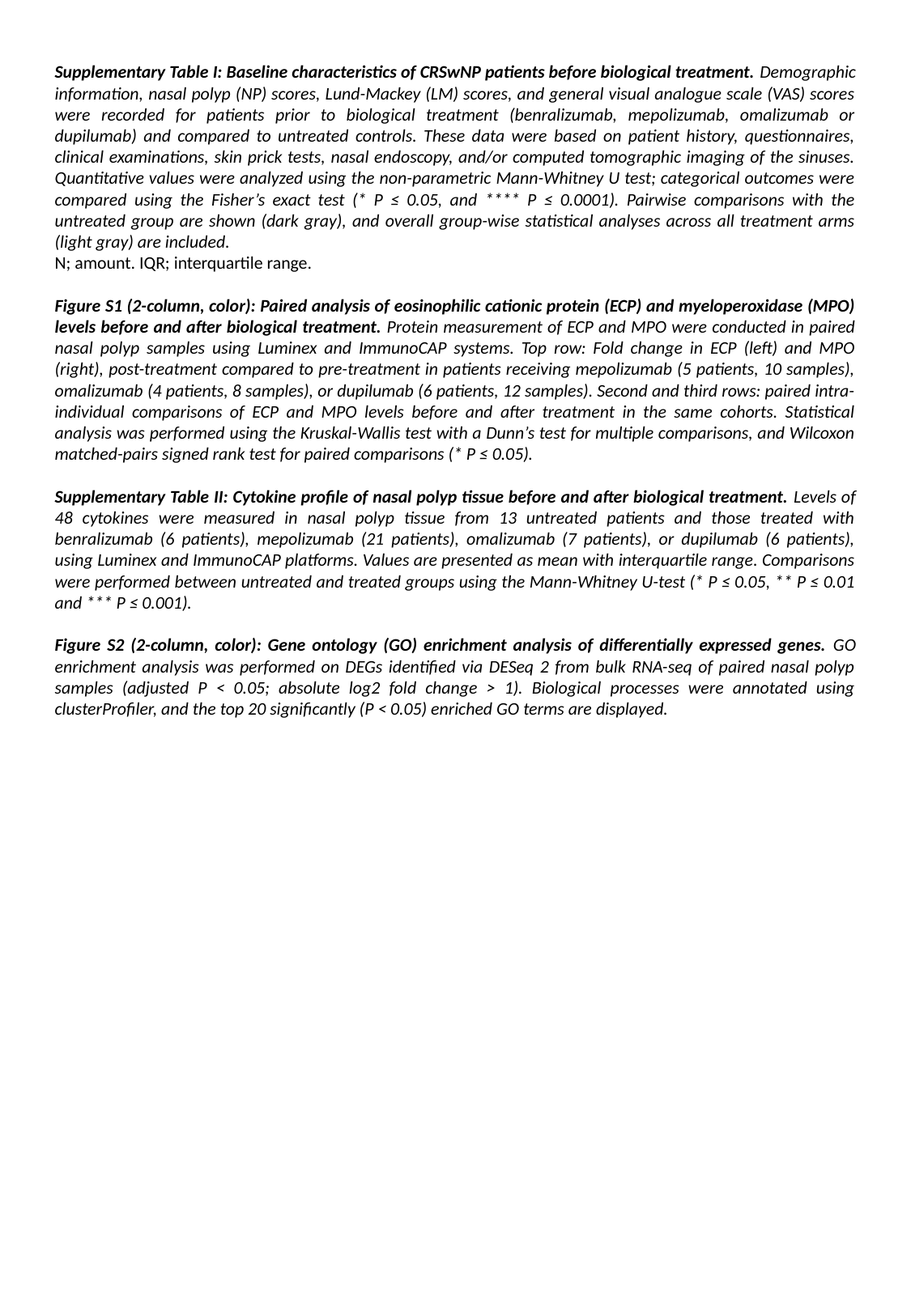

Supplementary Table I: Baseline characteristics of CRSwNP patients before biological treatment. Demographic information, nasal polyp (NP) scores, Lund-Mackey (LM) scores, and general visual analogue scale (VAS) scores were recorded for patients prior to biological treatment (benralizumab, mepolizumab, omalizumab or dupilumab) and compared to untreated controls. These data were based on patient history, questionnaires, clinical examinations, skin prick tests, nasal endoscopy, and/or computed tomographic imaging of the sinuses. Quantitative values were analyzed using the non-parametric Mann-Whitney U test; categorical outcomes were compared using the Fisher’s exact test (* P ≤ 0.05, and **** P ≤ 0.0001). Pairwise comparisons with the untreated group are shown (dark gray), and overall group-wise statistical analyses across all treatment arms (light gray) are included.
N; amount. IQR; interquartile range.
Figure S1 (2-column, color): Paired analysis of eosinophilic cationic protein (ECP) and myeloperoxidase (MPO) levels before and after biological treatment. Protein measurement of ECP and MPO were conducted in paired nasal polyp samples using Luminex and ImmunoCAP systems. Top row: Fold change in ECP (left) and MPO (right), post-treatment compared to pre-treatment in patients receiving mepolizumab (5 patients, 10 samples), omalizumab (4 patients, 8 samples), or dupilumab (6 patients, 12 samples). Second and third rows: paired intra-individual comparisons of ECP and MPO levels before and after treatment in the same cohorts. Statistical analysis was performed using the Kruskal-Wallis test with a Dunn’s test for multiple comparisons, and Wilcoxon matched-pairs signed rank test for paired comparisons (* P ≤ 0.05).
Supplementary Table II: Cytokine profile of nasal polyp tissue before and after biological treatment. Levels of 48 cytokines were measured in nasal polyp tissue from 13 untreated patients and those treated with benralizumab (6 patients), mepolizumab (21 patients), omalizumab (7 patients), or dupilumab (6 patients), using Luminex and ImmunoCAP platforms. Values are presented as mean with interquartile range. Comparisons were performed between untreated and treated groups using the Mann-Whitney U-test (* P ≤ 0.05, ** P ≤ 0.01 and *** P ≤ 0.001).
Figure S2 (2-column, color): Gene ontology (GO) enrichment analysis of differentially expressed genes. GO enrichment analysis was performed on DEGs identified via DESeq 2 from bulk RNA-seq of paired nasal polyp samples (adjusted P < 0.05; absolute log2 fold change > 1). Biological processes were annotated using clusterProfiler, and the top 20 significantly (P < 0.05) enriched GO terms are displayed.
